# Effects of Nasal Continuous Positive Airway Pressure and High-Flow Nasal Cannula on Sucking, Swallowing, and Breathing during Bottle-Feeding in Lambs

**DOI:** 10.3389/fped.2017.00296

**Published:** 2018-01-17

**Authors:** Nathalie Samson, Charlène Nadeau, Laurence Vincent, Danny Cantin, Jean-Paul Praud

**Affiliations:** ^1^Neonatal Respiratory Research Unit, Department of Pediatrics, Université de Sherbrooke, Sherbrooke, QC, Canada; ^2^Neonatal Respiratory Research Unit, Department of Pharmacology – Physiology, Université de Sherbrooke, Sherbrooke, QC, Canada

**Keywords:** sucking–swallowing–breathing coordination, nasal continuous positive airway pressure, high-flow nasal cannula, bottle-feeding efficiency and safety, full-term lambs

## Abstract

The use of prolonged respiratory support under the form of high-flow nasal cannula (HFNC) or nasal continuous positive airway pressure (nCPAP) is frequent in newborn infants. Introduction of oral feeding under such nasal respiratory support is, however, highly controversial among neonatologists, due to the fear that it could disrupt sucking, swallowing, and breathing coordination and in turn induce cardiorespiratory events. The recent observation of tracheal aspirations during bottle-feeding in preterm infants under nCPAP justifies the use of animal models to perform more comprehensive physiological studies on the subject, in order to gain further insights for clinical studies. The objective of this study was to assess and compare the impact of HFNC and nCPAP on bottle-feeding in newborn lambs, in terms of bottle-feeding efficiency and safety as well as sucking–swallowing–breathing coordination. Eight full-term lambs were instrumented to record sucking, swallowing, and respiration as well as electrocardiogram and oxygenation. Lambs were bottle-fed in a standardized manner during three randomly ordered conditions, namely nCPAP 6 cmH_2_O, HFNC 7 L/min, and no respiratory support. Results revealed that nCPAP decreased feeding duration [25 vs. 31 s (control) vs. 57 s (HFNC), *p* = 0.03] and increased the rate of milk transfer [2.4 vs. 1.9 mL/s (control) vs.1.1 mL/s (HFNC), *p* = 0.03]. No other indices of bottle-feeding safety or sucking–swallowing–breathing coordination were significantly altered by HFNC or nCPAP. In conclusion, our results obtained in full-term newborn lambs suggest that: (i) nCPAP 6 cmH_2_O, but not HFNC 7 L/min, increases bottle-feeding efficiency; (ii) bottle-feeding is safe under nCPAP 6 cmH_2_O and HFNC 7 L/min, with no significant alteration in sucking–swallowing–breathing coordination. The present informative and reassuring data in full-term healthy lambs must be complemented by similar studies in preterm lambs, including mild-to-moderate respiratory distress alleviated by respiratory support in order to mimic preterm infants with bronchopulmonary dysplasia and pave the way for clinical studies.

## Introduction

Nasal respiratory support (NRS), especially nasal continuous positive airway pressure (nCPAP) and high-flow nasal cannula (HFNC), has become the standard of care in convalescing preterm infants with bronchopulmonary dysplasia (BPD) and/or cardiorespiratory events (1–3). However, the prolonged use of NRS can delay the attainment of full oral feeding, which is a critical milestone for the preterm infant (4). Current knowledge strongly suggests that early introduction of oral feeding accelerates feeding maturation in preterm infants (5, 6). However, it is feared that nCPAP or HFNC can disrupt sucking–swallowing–breathing coordination and in turn induce cardiorespiratory events *via* laryngeal penetration and/or tracheal aspiration. Until now, the variable results and conflicting conclusions of the available clinical studies (7–12) have precluded the publication of evidence-based guidelines for feeding infants under NRS. Consequently, initiation of oral feeding in preterm infants under NRS remains a controversial topic among neonatologists (9, 11, 12).

Given the risk of tracheal aspiration in fragile preterm infants fed orally under nCPAP (11), we initiated a research program in newborn ovine models a few years ago with the aim of gaining new physiological knowledge on the effect of NRS on the precise coordination between sucking, swallowing, and breathing as well as on bottle-feeding efficiency and safety. We believe that observations in ovine models will ultimately help in designing safer clinical studies. As a result, we have previously shown that bottle-feeding under nCPAP is safe in both full-term (13) and preterm lambs (14) and is more efficient in preterm lambs (14), with no significant alteration in sucking–swallowing–breathing coordination. Given the increasing use of HFNC in neonates, it also becomes imperative to assess its impact on bottle-feeding. To our knowledge, this impact of HFNC has never been studied but our preliminary observations suggest that bottle-feeding efficiency is not supported by HFCN as well as by nCPAP.

The overarching aim of the present study was to assess the effect of HFNC and nCPAP on bottle-feeding in healthy full-term lambs. Thus, our main hypothesis was that nCPAP increases bottle-feeding efficiency and that this effect is less pronounced with HFNC. In addition, we hypothesized that bottle-feeding is safe under nCPAP or HFNC and that the latter does not alter sucking–swallowing–breathing coordination.

## Materials and Methods

This study was carried out in accordance with the recommendations of the Canadian Council on Animal Care. The protocol was approved by the Ethics Committee for Animal Care and Experimentation of the Université de Sherbrooke. Eight healthy, full-term, male mixed-bred lambs, aged 4–5 days, and weighing 3.0 ± 0.3 kg (mean ± SD) were included in the study.

### Chronic Instrumentation and Recording Equipment

Details of the instrumentation have previously been described (13, 14). Briefly, chronic surgical instrumentation was performed under general anesthesia (2% isoflurane) to insert: (i) custom-built bipolar electrodes into both thyroarytenoid muscles (laryngeal constrictors) for recording swallowing activity; (ii) a catheter into the left carotid artery for measuring arterial blood gases; and (iii) a transcutaneous catheter to monitor tracheal pressure variations as a marker of respiration.

Instrumentation was completed immediately before recordings with (i) subcutaneous needle electrodes for electrocardiogram recordings; (ii) elastic bands on the chest and abdomen to monitor lung volume variations semiquantitatively *via* respiratory inductance plethysmography; and (iii) a pulse oximetry probe placed at the base of the tail for continuous monitoring of oxygen hemoglobin saturation (SpO_2_). In addition, a pressure catheter was introduced into the bottle teat to monitor sucking (positive expression amplitude).

Nasal continuous positive airway pressure was delivered with the Infant Flow nCPAP system, delivering a variable-flow CPAP of 6 cmH_2_O (Cardinal Health, Dublin, OH, USA) through a plastic nasal mask custom-built for newborn lambs. The mask included two short nasal cannulae and was secured with a headgear. The interior of the mask was filled with dental paste to decrease dead space (<2 mL) and prevent leaks. The mask was installed on the lamb’s muzzle in such a manner that the lamb was able to open its mouth at will and readily drink from a bottle. HFNC was delivered using the Optiflow system with intermediate infant-size nasal cannulae (BC2755, Fisher & Paykel, Mississauga, ON, Canada) along with delivery of maximum flow available with this equipment (7 L/min). The levels of nCPAP and HFNC were selected on the basis of those reported in newborn infants, i.e., 5–7 cmH_2_O for nCPAP and 2–8 L/min for HFNC (15). Physiological signals were transmitted wirelessly with our custom-designed radiotelemetry system (16) and continuously recorded on a PC using AcqKnowledge software (version 4.1 Biopac Systems Canada, Montreal, QC, Canada). The entire recording period was video recorded, and an experimenter noted all events occurring during the recordings.

### Design of the Study

All lambs were separated from their mother upon arrival in our animal quarters and taught to feed from a bottle by an animal care technician. The lambs were placed in a Plexiglas chamber between experiments and were able to feed *ad libitum* from a custom-built lamb feeder. Only reconstituted ewe milk was given to the lambs throughout the study.

Following surgical instrumentation, a 48-h period was allowed to recover from pain and anesthesia. Lambs used in the present study were also involved in another study aiming at comparing the effects of NRS on the number of gastroesophageal refluxes (unpublished results). Three randomly ordered conditions (blocked randomization), namely nCPAP 6 cmH_2_O, HFNC 7 L/min, and no respiratory support (control), were studied on three consecutive days in each lamb using a crossover design. Hence, on each experimental morning, the lambs first underwent a 6 h polysomnographic recording with either nCPAP, HFNC, or no respiratory support to assess gastroesophageal reflux *via* a nasoesophageal catheter. A 5-min pause was allowed for bottle-feeding at mid-time recording, hence 3 h before the present study on bottle-feeding.

Standardized bottle-feeding was performed immediately after the end of the 6 h polysomnographic recording, at around 2:00 p.m. While still under nCPAP, HFNC or in control condition, the lambs were comfortably positioned in a sling with loose restraints and offered a bottle filled with 60 mL of ewe milk heated at 39°C, corresponding to the usual amount ingested from a bottle by a full-term newborn lamb. The bottle was offered to the lambs a maximum of three times, after which the feeding session was considered over. Reasons to discontinue feeding were mainly agitation by the lamb or refusal to drink. Recordings were continued under NRS for 15 min after bottle-feeding. Arterial blood samples were taken immediately before feeding and 1, 5, and 15 min after feeding completion.

### Data Analysis

The raw EMG signals were rectified, integrated, and moving time averaged (100 ms). All signals were analyzed before (20 s baseline), during (total length of bottle-feeding duration), and after (first 30 s following bottle-feeding) bottle-feeding in each condition (control, nCPAP, and HFNC) (14) in the eight lambs.

Tracheal pressure recording was used to measure the end-expiratory positive pressure applied to the respiratory system by nCPAP and HFNC and to calculate an index of the work of breathing in each of the three conditions. Hence, the product of the amplitude of the decrease in tracheal pressure during inspiration and the respiratory rate was calculated and averaged over 10 respiratory cycles during the baseline preceeding recording.

Bottle-feeding efficiency was quantified by feeding duration (s), the average rate of milk transfer (mL/s), the total number of sucks (SU) and swallows (SW), and the mean positive pressure in the teat. The safety of bottle-feeding was quantified by the number of heart decelerations [defined by a decrease in heart rate (HR) of at least 33%, regardless of the duration], minimal HR (min^−1^), percentage decrease in HR, total duration of cardiac inhibition (total time spent in heart decelerations), minimal SpO_2_ (%), percentage decrease in SpO_2_ (%), and number of coughs during and within 30 s following bottle-feeding. Sucking–swallowing–breathing coordination was quantified by the percentage of feeding duration spent in apnea (defined as at least two missed breaths) and the percentage of SW occurring during an apnea. The rhythmic stability of feeding was quantified by the time interval between SU–SU, SU–SW, SW–SW, and SW–breath (BR), as well as by the coefficient of variation (COV) (17) of SU–SU, SU–SW, SW–SW, and SW–BR. A lower COV value indicates a more stable rhythm.

### Statistical Analysis

The statistical power was calculated using the GLIMMPSE software (http://glimmpse.samplesizeshop.org), which allows taking into account the repeated measures design of our study. In a model comprised of three experimental conditions, repeated measures, an unstructured covariance structure, and a within-subject correlation of 0.4, our sample of eight lambs had a statistical power of 95% to detect a difference of 0.5 SD within a normal *Z*-distribution of the main outcome variable (feeding duration) using a two-tailed test with an alpha error of 5%.

Results were first averaged for each lamb, then averaged for each condition (control, nCPAP, and HFNC). Values are expressed as median (Q1, Q3). The Friedman test completed by the Wilcoxon signed-rank test when appropriate was used to compare the experimental conditions. All statistical analyses were performed with SPSS. A *p* < 0.05 was considered statistically significant.

## Results

**Figure 1:**
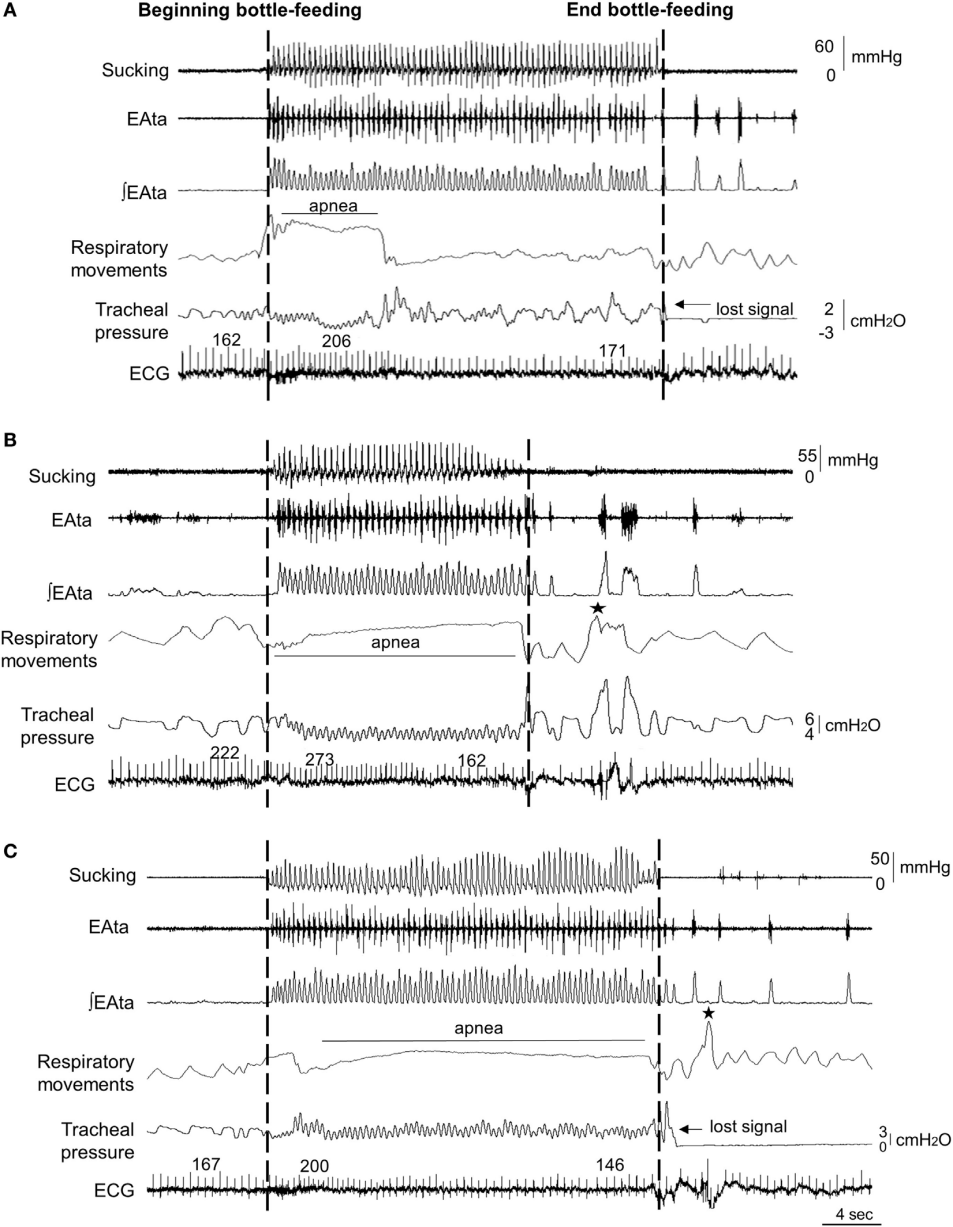
Sample tracings of bottle-feeding during no respiratory support (control condition) **(A)**, nasal continuous positive airway pressure (nCPAP) 6 cmH_2_O **(B)** and high-flow nasal cannula (HFNC) 7 L/min **(C)** in one full-term lamb, showing decreased feeding duration with nCPAP 6 cmH_2_O only. Respiratory inhibition (apnea) was unusually pronounced during nCPAP and HFNC in this particular lamb compared to all the other lambs. The two dashed lines indicate the bottle-feeding period. Abbreviations from top to bottom: sucking (positive expression pressure on the bottle teat); EAta, electrical activity of the thyroarytenoid muscle for recording swallowing activity; (∫EAta, moving time averaged EAta; respiratory movements, sum signal of the respiratory inductance plethysmography; ECG, electrocardiogram; and ★, body movements. Of note, the oxygen hemoglobin saturation signal was not adequate in this lamb due to dark pigmentation of the tail.

The median end-expiratory tracheal pressure during control condition, nCPAP 6 cmH_2_O, and HFNC 7 L/min was 0.2 (0.1, 0.4), 5 (5, 6), and 2 (1, 2) cmH_2_O, respectively (Table 1). In addition, compared to control condition [110 (93,161) cmH_2_O/min], work of breathing was significantly decreased by nCPAP [62 (51, 79) cmH_2_O/min, *p* = 0.01] but not by HFNC [119 (85, 176) cmH_2_O/min, *p* = 0.6]. Figure 1 illustrates a sample tracing of bottle-feeding during the three experimental conditions.

**Table 1:** Effect of nCPAP and HFNC on the efficiency, safety, and rhythmic stability of feeding in full-term lambs.

	Control, *n* = 8	nCPAP 6 cmH_2_O, *n* = 8	HFNC 7 L/min, *n* = 8
End-expiratory tracheal pressure (cmH_2_O)	0.2 (0.1, 0.4)	5 (5, 6)*^,^^‡^	2 (1, 2)*
**Efficiency**			
Feeding duration (s)	31 (28, 40)	25 (16, 34)*^,^^‡^	57 (31, 68)
Rate of milk transfer (mL/sec)	1.9 (1.5, 2.2)	2.4 (1.9, 3.8)*^,^^‡^	1.1 (0.9, 2.0)
Expression amplitude (mmHg)	+32 (26, 45)	+34 (30, 41)	+24 (20, 38)
Total number of SU	68 (58, 90)	52 (46, 67)	69 (66, 83)
Total number of SW	81 (69, 103)	67 (51, 83)	88 (82, 98)
**Safety**			
Number of coughs	0 (0, 0)	0 (0, 0)	0 (0, 0)
Number of cardiac decelerations	5 (0, 10)	0 (0, 4)	1 (0, 3)
Minimal HR	85 (71, 121)	138 (79, 186)	109 (93, 147)
% decrease in HR	53 (42, 61)	26 (23, 55)	33 (31, 59)
Duration of cardiac inhibition (s)	5 (0, 11)	0 (0, 3)	0 (0, 3)
Minimal SpO_2_ (%)	85 (78, 92)	92 (89, 95)	91 (84, 93)
% decrease in SpO_2_	5 (4, 14)	1 (1, 4)	4 (2, 8)
**SU–SW–BR coordination**			
% feeding time spent in apnea	18 (16, 42)	38 (24, 70)	19 (13, 37)
% SW occurring during apnea	22 (19, 57)	53 (28, 72)	30 (22, 60)
SU–SU covariance	0.8 (0.4, 1)	0.1 (0.1, 0.8)	1.2 (0.4, 2)
SU–SU interval (s)	0.4 (0.4, 0.4)	0.3 (0.3, 0.5)	0.6 (0.3, 0.9)
SU–SW covariance	0.2 (0.2, 0.3)	0.2 (0.09, 0.2)	0.3 (0.2, 0.4)
SU–SW interval (s)	0.2 (0.2, 0.3)	0.2 (0.1, 0.2)	0.3 (0.1, 0.4)
SW–SW covariance	0.4 (0.3, 0.6)	0.3 (0.2, 0.5)	0.7 (0.4, 1)
SW–SW interval (s)	0.4 (0.4, 0.4)	0.3 (0.3, 0.4)	0.5 (0.3, 0.7)
SW–BR covariance	0.8 (0.7, 0.9)	0.8 (0.7, 0.9)	0.9 (0.7, 1)
SW–BR interval (s)	1.6 (1.3, 2.7)	2.4 (1.4, 4)	2.4 (1.4, 4.5)

*Results are presented as median (Q1, Q3)*.

*Cardiac deceleration was defined by a % decrease in HR ≥ 33%, regardless of the duration*.

*No deceleration longer than 5 s was noted*.

*p < 0.05 vs. control.

*^‡^p < 0.05 vs. HFNC*.

*nCPAP, nasal continuous positive airway pressure 6 cmH_2_O; HFNC, high-flow nasal cannula 7 L/min; SU, sucking; SW, swallowing; HR, heart rate; SpO_2_, arterial hemoglobin saturation in oxygen; BR, breathing; covariance, coefficient of variation of the time interval between SU, SW, and BR*.

### Efficiency of Bottle-Feeding under nCPAP and HFNC

Nasal continuous positive airway pressure increased bottle-feeding efficiency by decreasing feeding duration [25 vs. 31 s (control) vs. 57 s (HFNC), *p* = 0.03] and increasing the rate of milk transfer [2.4 vs. 1.9 mL/s (control) vs.1.1 mL/s (HFNC), *p* = 0.03] compared to control and HFNC (Table 1). No other indices of bottle-feeding efficiency were altered by nCPAP or HFNC. Of note, results in one lamb greatly differed from those of all the other lambs. Removal of this animal data revealed that nCPAP further increased bottle-feeding efficiency by decreasing feeding duration, lowering the number of sucks and swallows necessary to drink the entire bottle, and increasing the rate of milk transfer (*p* between 0.02 and 0.008).

### Safety of Bottle-Feeding under nCPAP and HFNC

No statistical differences were noted between groups for any of the cardiorespiratory variables during bottle-feeding (Table 1). A decreased respiratory rate with nCPAP at baseline (27 vs. 42 min^−1^, *p* = 0.004), as well as at 5 (32 vs. 48 min^−1^, *p* = 0.02) and 15 min (33 vs. 53 min^−1^, *p* = 0.01) after bottle-feeding was observed compared to control condition (Table 2). Conversely, PaO_2_ was increased with nCPAP (Table 2) and, if anything, minimal SpO_2_ was higher (Table 2) before and 5 min after bottle-feeding, while minimal SpO_2_ remained unchanged during bottle-feeding (Table 1). In comparison, HFNC did not alter respiratory rate at baseline and did not lead to a significant decrease in respiratory rate after bottle-feeding. Finally, although no coughs were observed in any of the lambs during bottle-feeding, one single lamb under nCPAP presented two coughs within the 10 s following bottle removal.

**Table 2:** Cardiorespiratory variables and arterial blood gases before and following bottle-feeding in full-term lambs.

	Control, *n* = 8	nCPAP 6 cmH_2_O, *n* = 8	HFNC 7 L/min, *n* = 8
**Before bottle-feeding**							
Heart rate (bpm)	201 (175, 211)	216 (193, 230)	224 (195, 251)
Respiratory rate (min^−1^)	42 (36, 57)	27 (24, 36)*	42 (35, 54)
SpO_2_ (%)	95 (94, 95)	95 (95, 97)	96 (94, 98)
PaO_2_ (mmHg)	82 (74, 91)	94 (83, 108)*^,^^‡^	88 (73, 94)
PaCO_2_ (mmHg)	40 (37, 41)	42 (39, 45)	40 (38, 43)
pH	7.45 (7.44, 7.46)	7.44 (7.42, 7.45)	7.45 (7.44, 7.46)
**1 min after bottle-feeding**											
Heart rate (bpm)	190 (173, 206)	209 (192, 235)	230 (193, 264)
Respiratory rate (min^−1^)	42 (35, 44)	32 (26, 39)	42 (34, 54)
SpO_2_ (%)	95 (95, 96)	95 (93, 97)	95 (92, 95)
PaO_2_ (mmHg)	91 (83, 96)	98 (91, 108)	83 (73, 97)
PaCO_2_ (mmHg)	40 (36, 42)	39 (37, 40)	40 (40, 43)
pH	7.43 (7.43, 7.45)	7.43 (7.42, 7.44)	7.44 (7.41, 7.44)
**5 min after bottle-feeding**											
Heart rate (bpm)	201 (177, 229)	235 (214, 245)	232 (190, 251)
Respiratory rate (min^−1^)	48 (41, 67)	32 (29, 42)*	36 (32, 44)
SpO_2_ (%)	95 (92, 96)	93 (91, 96)	94 (93, 95)
PaO_2_ (mmHg)	82 (76, 88)	90 (78, 103)*^,^^‡^	77 (69, 100)
PaCO_2_ (mmHg)	41 (37, 42)	40 (38, 42)	40 (34, 41)
pH	7.43 (7.42, 7.45)	7.43 (7.41, 7.44)	7.44 (7.43, 7.45)
**15 min after bottle-feeding**											
Heart rate (bpm)	199 (180, 214)	233 (218, 242)	234 (196, 265)
Respiratory rate (min^−1^)	53 (45, 57)	33 (29, 39)*	42 (32, 65)
SpO_2_ (%)	95 (94, 96)	95 (93, 96)	92 (91, 95)
PaO_2_ (mmHg)	86 (82, 89)	87 (82, 99)	85 (64, 88)
PaCO_2_ (mmHg)	41 (40, 43)	42 (40, 44)	39 (37, 42)
pH	7.44 (7.43, 7.45)	7.43 (7.41, 7.45)	7.45 (7.44, 7.45)

*Results are presented as median (Q1, Q3)*.

*p < 0.05 vs. control.

*^‡^p < 0.05 vs. HFNC*.

*nCPAP, nasal continuous positive airway pressure 6 cmH_2_O; HFNC, high-flow nasal cannula 7 L/min; SpO_2_, hemoglobin saturation; PaO_2_, partial pressure of oxygen in arterial blood; PaCO_2_, partial pressure of carbon dioxide in arterial blood*.

### Sucking–Swallowing–Breathing Coordination under nCPAP and HFNC

Both nCPAP and HFNC had no significant effect on mean data with regard to the coordination between sucking, swallowing, and breathing (Table 1). However, further analysis revealed that compared to controls, the percentage of feeding spent in apnea was increased in five and three lambs during nCPAP and HFNC, respectively. Of note, after removal of the aforementioned “outlier lamb,” results revealed an improvement in sucking–swallowing coordination with nCPAP compared to HFNC (*p* = 0.08).

## Discussion

The present study revealed that neither nCPAP 6 cmH_2_O nor HFNC 7 L/min altered the safety of bottle-feeding and the coordination between sucking–swallowing–breathing in full-term lambs. In addition, nCPAP, but not HFNC, increased bottle-feeding efficiency. These results constitute the first detailed physiological assessment of the effects of nCPAP and HFNC on bottle-feeding in the neonatal period.

### Literature Data on Oral Feeding under NRS

Oral feeding in preterm infants under prolonged NRS is currently highly debated. The lack of evidence-based guidelines on oral feeding in preterm infants under nCPAP and HFNC is due to the paucity of available data. A few recent studies have shown that initiation of oral feeding can be successful under nCPAP (9, 12) or HFNC (10, 12, 18). However, data comparing oral feeding under nCPAP and HFNC are nearly inexistent. Preterm infants with BPD were recently shown to achieve full oral feeding sooner when supported by nCPAP followed by HFNC vs. prolonged nCPAP alone. However, this was likely due to the fact that oral feeding was attempted, if deemed appropriate, under HFNC but never under nCPAP (18). Accordingly, another study did not find any difference between nCPAP and HFNC on the time needed to attain full oral feeding (12). As recently highlighted, there are no formal data documenting whether the presence of HFNC impairs swallowing function during suckle feeding or promotes tracheal aspiration, which led the authors to determinedly advise against the thoughtless use of oral feeding under HFNC before further information is available (19).

### Efficiency of Bottle-Feeding under nCPAP and HFNC

The present results confirm that bottle-feeding efficiency is improved in full-term lambs under nCPAP, as recently shown in preterm lambs (14). This improvement in bottle-feeding under nCPAP may be related to increased functional residual capacity (20), which promotes better oxygenation, as well as to the observed decreased work of breathing.

In contrast, bottle-feeding efficiency was not modified by HFNC compared to control conditions. We believe this is likely related to the absence of a distending pressure and/or a decrease in the work of breathing with HFNC as shown herein. In healthy adult volunteers, swallowing frequency was similarly found to be unaffected by HFNC (21). Future studies comparing the effects of nCPAP and HFNC at the same pharyngeal pressure, i.e., using nasal cannulae with lower leaks and/or a higher flow, are necessary to confirm the abovementioned hypothesis.

### Safety of Bottle-Feeding under nCPAP and HFNC

The safety of bottle-feeding while on prolonged NRS continues to raise many questions due to the discrepant available data. On the one hand, preterm infants under nCPAP can initiate pharyngeal reflexive swallowing as well as their room air-breathing counterparts (22), and oral feeding is possible in stable preterm infants under nCPAP or HFNC without apparent adverse outcomes (9, 12). In addition, HFNC enhances swallowing function by reducing the reflex latency in adult volunteers (21). On the other hand, a recent videofluoroscopic study in preterm infants concluded that nCPAP alters pharyngeal swallows and increases the risk of tracheal aspiration during oral feeding (11). Finally, despite the overall conclusion that oral feeding under HFNC was safe in infants with acute viral bronchiolitis, a few feeding-related adverse events were nonetheless observed (23, 24).

In the present study, bottle-feeding under nCPAP or HFNC did not increase cardiorespiratory events compared to control conditions. On the contrary, while not significant, hemoglobin desaturations appeared less marked with nCPAP. Of note, however, the presence of two isolated coughs (without any cardiorespiratory event) in a single lamb a few seconds after the end of bottle-feeding potentially raises the possibility of laryngeal penetration or tracheal aspiration. Identical observations have also been made on occasion in a few preterm lambs (14). Videofluoroscopic studies are needed in preterm lambs to confirm the presence or not of tracheal aspiration as well as to further understand and prevent the latter if present.

### Coordination between Sucking, Swallowing, and Breathing during Bottle-Feeding

Overall, both HFNC and nCPAP did not significantly decrease the coordination between sucking–swallowing–breathing. This is similar to previous observations on swallowing–breathing coordination in lambs under nCPAP (13) and in adult healthy volunteers under HNFC (21). Of note, tentative removal of one outlier lamb suggests that sucking–swallowing coordination is better with nCPAP vs. HFNC. Moreover, although not statistically significant, the time spent in apnea and the swallowing activity during apnea appeared to be increased during nCPAP, similar to that previously observed in preterm lambs (14). There are nevertheless differences between full-term and preterm lambs, with less than 25% of swallows found to occur during apnea in control conditions in the former vs. 50% in the latter (14). Also, the time spent in apnea during bottle-feeding under nCPAP is shorter in full-term than preterm lambs, maybe due to a stronger inhibitory effect of swallowing and nCPAP on breathing in preterms. Finally, while not statistically different, the suck–suck rhythm (SU–SU COV) analyzed herein was more stable during nCPAP and more variable under HFNC, albeit without any clear explanation.

### Limitations of the Study

As previously stated, the present physiological study aimed to assess and compare the impact of HFNC 7 L/min and nCPAP 6 cmH_2_O on nutritive swallowing and sucking–swallowing–breathing coordination in an ovine model. Limitations must be recognized with regard to the clinical relevance in infants requiring prolonged NRS. First, study was conducted in healthy full-term lambs, which do not fully represent the majority of (preterm) infants on NRS. This was, however, a deliberate choice to first gain knowledge in a full-term model before conducting future studies in preterm lambs, similar to that performed previously (14). Moreover, the recent publications on full-term infants bottle-fed under HFNC for acute viral bronchiolitis (23, 24) clearly highlight that new knowledge beyond preterm infants is needed. Second, while NRS is frequently used for treating mild-to-moderate BPD, the present study was performed in healthy lambs. Additional studies are thus needed in preterm lambs with mild-to-moderate respiratory distress alleviated by NRS. Finally, as already raised, the absence of videofluoroscopic assessment implies that silent tracheal aspirations may have been missed (11).

## Conclusion

Our results obtained in newborn lambs suggest that bottle-feeding is safe under nCPAP 6 cmH_2_O and HFNC 7 L/min and does not significantly alter sucking–swallowing–breathing coordination. In addition, while HFNC does not impede bottle-feeding, nCPAP increases its efficiency. Further studies with videoscopic assessment are needed in preterm lambs, without and with mild respiratory distress, i.e., in conditions closer to those encountered in the neonatal intensive care unit. Additional studies are also warranted to compare the impact of HFNC and nCPAP on bottle-feeding at the same distending tracheal pressure in order to gain sufficient physiological knowledge to pave the way for safer clinical studies in preterm infants.

## Ethics Statement

This study was carried out in accordance with the recommendations of the Canadian Council on Animal Care. The protocol was approved by the Ethics Committee for Animal Care and Experimentation of the Université de Sherbrooke.

## Author Contributions

NS and J-PP conceived and designed the study. NS, CN, LV, and DC performed the animal experiments. NS, CN, and LV analyzed the data. NS and J-PP interpreted the results obtained. NS drafted the manuscript. NS and J-PP revised the manuscript. All authors read and approved the final version of the manuscript and agreed to be accountable for all aspects of the work.

## Conflict of Interest Statement

The authors declare that the research was conducted in the absence of any commercial or financial relationships that could be construed as a potential conflict of interest.

## Abbreviations

NRS, nasal respiratory support; nCPAP, nasal continuous positive airway pressure; HFNC, high-flow nasal cannula; BPD, bronchopulmonary dysplasia.

## References

[1] DeMauro SB Millar D Kirpalani H. Noninvasive respiratory support for neonates. Curr Opin Pediatr (2014) 26(2):157–62. 10.1097/MOP.0000000000000066 24632541

[2] Di Fiore JM Poets CF Gauda E Martin RJ MacFarlane P. Cardiorespiratory events in preterm infants: interventions and consequences. J Perinatol (2016) 36(4):251–8. 10.1038/jp.2015.165 26583943

[3] Wilkinson D Andersen C O’Donnell CP De Paoli AG Manley BJ. High flow nasal cannula for respiratory support in preterm infants. Cochrane Database Syst Rev (2016) 2:CD006405. 10.1002/14651858.CD006405.pub3 26899543 PMC9371597

[4] American Academy of Pediatrics, Committee on Fetus and Newborn. Hospital discharge of the high-risk neonate. Pediatrics (2008) 122(5):1119–26. 10.1542/peds.2008-2174 18977994

[5] Simpson C Schanler RJ Lau C. Early introduction of oral feeding in preterm infants. Pediatrics (2002) 110(3):517–22. 10.1542/peds.110.3.517 12205253

[6] Pickler RH Chiarinai C Reyna BB. Relationship of the first suck burst to feeding outcomes in preterm infants. J Perinat Neonatal Nurs (2006) 20(2):157–62. 10.1097/00005237-200604000-00010 16714916 PMC3640461

[7] Bonner KM Mainous RO. The nursing care of the infant receiving bubble CPAP therapy. Adv Neonatal Care (2008) 8(2):78–95; quiz 96–77. 10.1097/01.ANC.0000317256.76201.72 18418205

[8] Maastrup R Bojesen SN Kronborg H Hallstrom I. Breastfeeding support in neonatal intensive care: a national survey. J Hum Lact (2012) 28(3):370–9. 10.1177/0890334412440846 22674965

[9] Hanin M Nuthakki S Malkar MB Jadcherla SR. Safety and efficacy of oral feeding in infants with BPD on nasal CPAP. Dysphagia (2015) 30(2):121–7. 10.1007/s00455-014-9586-x 25380678 PMC4800480

[10] Leder SB Siner JM Bizzarro MJ McGinley BM Lefton-Greif MA. Oral alimentation in neonatal and adult populations requiring high-flow oxygen via nasal cannula. Dysphagia (2016) 31(2):154–9. 10.1007/s00455-015-9669-3 26590570

[11] Ferrara L Bidiwala A Sher I Pirzada M Barlev D Islam S Effect of nasal continuous positive airway pressure on the pharyngeal swallow in neonates. J Perinatol (2017) 37(4):398–403. 10.1038/jp.2016.229 28055023

[12] Glackin SJ O’Sullivan A George S Semberova J Miletin J. High flow nasal cannula versus NCPAP, duration to full oral feeds in preterm infants: a randomised controlled trial. Arch Dis Child Fetal Neonatal Ed (2017) 102(4):F329–32. 10.1136/archdischild-2016-311388 28011792

[13] Bernier A Catelin C Ahmed MA Samson N Bonneau P Praud JP. Effects of nasal continuous positive-airway pressure on nutritive swallowing in lambs. J Appl Physiol (1985) (2012) 112(12):1984–91. 10.1152/japplphysiol.01559.2011 22500003

[14] Samson N Michaud A Othman R Nadeau C Nault S Cantin D Nasal continuous positive airway pressure influences bottle-feeding in preterm lambs. Pediatr Res (2017) 82(6):926–33. 10.1038/pr.2017.162 28700565

[15] Sinha IP McBridge AKS Smith R Fernandes RM. CPAP and high-flow nasal cannula oxygen in bronchiolitis. Chest (2015) 148(3):810–23. 10.1378/chest.14-1589 25836649

[16] Samson N Dumont S Specq ML Praud JP. Radio telemetry devices to monitor breathing in non-sedated animals. Respir Physiol Neurobiol (2011) 179(2–3):111–8. 10.1016/j.resp.2011.09.008 21964163

[17] Gewolb IH Fishman D Qureshi MA Vice FL. Coordination of suck-swallow-respiration in infants born to mothers with drug-abuse problems. Dev Med Child Neurol (2004) 46(10):700–5. 10.1017/S0012162204001173 15473175

[18] Shetty S Hunt K Douthwaite A Athanasiou M Hickey A Greenough A. High-flow nasal cannula oxygen and nasal continuous positive airway pressure and full oral feeding in infants with bronchopulmonary dysplasia. Arch Dis Child Fetal Neonatal Ed (2016) 101(5):F408–11. 10.1136/archdischild-2015-309683 26883075

[19] Dodrill P Gosa M Thoyre S Shaker C Pados B Park J FIRST, DO NO HARM: a response to “oral alimentation in neonatal and adult populations requiring high-flow oxygen via nasal cannula”. Dysphagia (2016) 31(6):781–2. 10.1007/s00455-016-9722-x 27435249

[20] Saunders RA Milner AD Hopkin IE. The effects of continuous positive airway pressure on lung mechanics and lung volumes in the neonate. Biol Neonate (1976) 29(3–4):178–86. 10.1159/000240862 782570

[21] Sanuki T Mishima G Kiriishi K Watanabe T Okayasu I Kawai M Effect of nasal high-flow oxygen therapy on the swallowing reflex: an in vivo volunteer study. Clin Oral Investig (2017) 21(3):915–20. 10.1007/s00784-016-1822-3 27055846

[22] Jadcherla SR Hasenstab KA Sitaram S Clouse BJ Slaughter JL Shaker R. Effect of nasal noninvasive respiratory support methods on pharyngeal provocation-induced aerodigestive reflexes in infants. Am J Physiol Gastrointest Liver Physiol (2016) 310(11):G1006–14. 10.1152/ajpgi.00307.2015 27012774 PMC4935482

[23] Slain KN Martinez-Schlurmann N Shein SL Stormorken A. Nutrition and high-flow nasal cannula respiratory support in children with bronchiolitis. Hosp Pediatr (2017) 7(5):256–62. 10.1542/hpeds.2016-0194 28424243

[24] Sochet AA McGee JA October TW. Oral nutrition in children with bronchiolitis on high-flow nasal cannula is well tolerated. Hosp Pediatr (2017) 7(5):249–55. 10.1542/hpeds.2016-0131 28424245

